# Short‐course intrapleural alteplase and DNase in complex effusion with bleeding risk

**DOI:** 10.1002/rcr2.648

**Published:** 2020-08-25

**Authors:** Xiong Khee Cheong, Andrea Yu‐Lin Ban, Mohamed Faisal Abdul Hamid

**Affiliations:** ^1^ Respiratory Unit University Kebangsaan Malaysia Medical Centre Kuala Lumpur Malaysia

**Keywords:** Alteplase, DNase, empyema, intrapleural fibrinolysis, pleural infection

## Abstract

Pleural infection is an important clinical problem with significant morbidity. In poorly draining complex pleural effusions, the current management favours a less invasive image‐guided placement of smaller bore catheters and adjunctive intrapleural fibrinolysis therapy (IPFT). We describe our experience of using IPFT in three patients with different bleeding risks with complex pleural effusions. The first was a 30‐year‐old with transfusion‐dependent β‐thalassemia with haemoglobin of 7.8 g/dL; second was an 87‐year‐old on dabigatran with haemoglobin of 10 g/dL; and the third was an 80‐year‐old with diffuse large B‐cell lymphoma with haemoglobin of 8.6 g/dL. All three patients received three doses of alteplase and deoxyribonuclease (DNase) without any adverse effects of bleeding and had resolution of the effusion. This case series is an addition to the current literature on the safety of IPFT and we highlight the use of IPFT in patients with low baseline haemoglobin and on anticoagulation therapy.

## Graphical abstract

We described three patients with complex pleural effusions who received intrapleural fibrinolysis therapy with different bleeding risks.

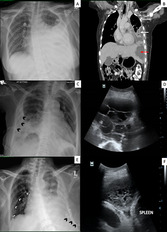

## Introduction

Empyema and complex pleural effusion cause significant morbidity and mortality in up to 40% of hospitalized patients with bacterial pneumonia [1]. The mainstay of therapy is antibiotics and drainage of the infected pleural fluid. The use of intrapleural fibrinolysis in empyema was first described in 1949 using streptococcal fibrinolysin and desoxyribonuclease [2]. More recent data suggest the benefit of a less invasive approach with intrapleural fibrinolysis therapy (IPFT) in poorly resolving complex effusions over surgical intervention. Multi‐center Intra‐pleural Sepsis Trial (MIST‐2) demonstrated improved pleural drainage with the combination of intrapleural alteplase 10 mg and dornase alfa (Pulmozyme, Hoffmann‐La Roche Ltd) 5 mg [3]. We report three patients with low haemoglobin with different bleeding risk, successfully treated with a modified regimen of three doses of intrapleural alteplase 16 mg and DNase 5 mg. The rationale of using 16 mg alteplase was determined by the alteplase formulation of 50 mg in our country. In this case series, we demonstrated the successful outcome and safety of this modified regimen. Demographic and clinical characteristics of the patients are displayed in Table 1.

**Table 1:** Demographic and clinical characteristics of the patients.

Demographic data		Case 1	Case 2	Case 3
Age (years)		30	87	80
Sex		Female	Female	Female
Outcome of effusion		Complete resolution	Complete resolution	Complete resolution
Blood parameters				
Haemoglobin (g/dL)	Admission	7.4	10.0	8.6
	Discharge	8.0	10.2	7.8
White cell count (×10^9/^ L)	Admission	24.4	6.4	6.7
	Discharge	19.2	6.0	9.7
C‐reactive protein (mg/dL)	Admission	7.38	1.83	17.15
	Discharge	0.48	0.32	0.87
LDH (U/L)		Not available	140	Not available
Protein (g/dL)		60	72	28
Pleural fluid analysis				
Site of effusion		Left	Right	Left
LDH (U/L)		750	434	1561
Protein (g/dL)		72	36	28
Glucose (mmol/L)		<0.3	5.6	<0.3
Pleural catheter				
Intercostal chest catheter size (Fr)		20	14	8
Pleural drainage post intrapleural fibrinolysis (mL)				
Day 1		280	180	510
Day 2		380	1750	900
Day 3		300	50	800

LDH, lactate dehydrogenase.

## Case Series

### Case 1

**Figure 1:**
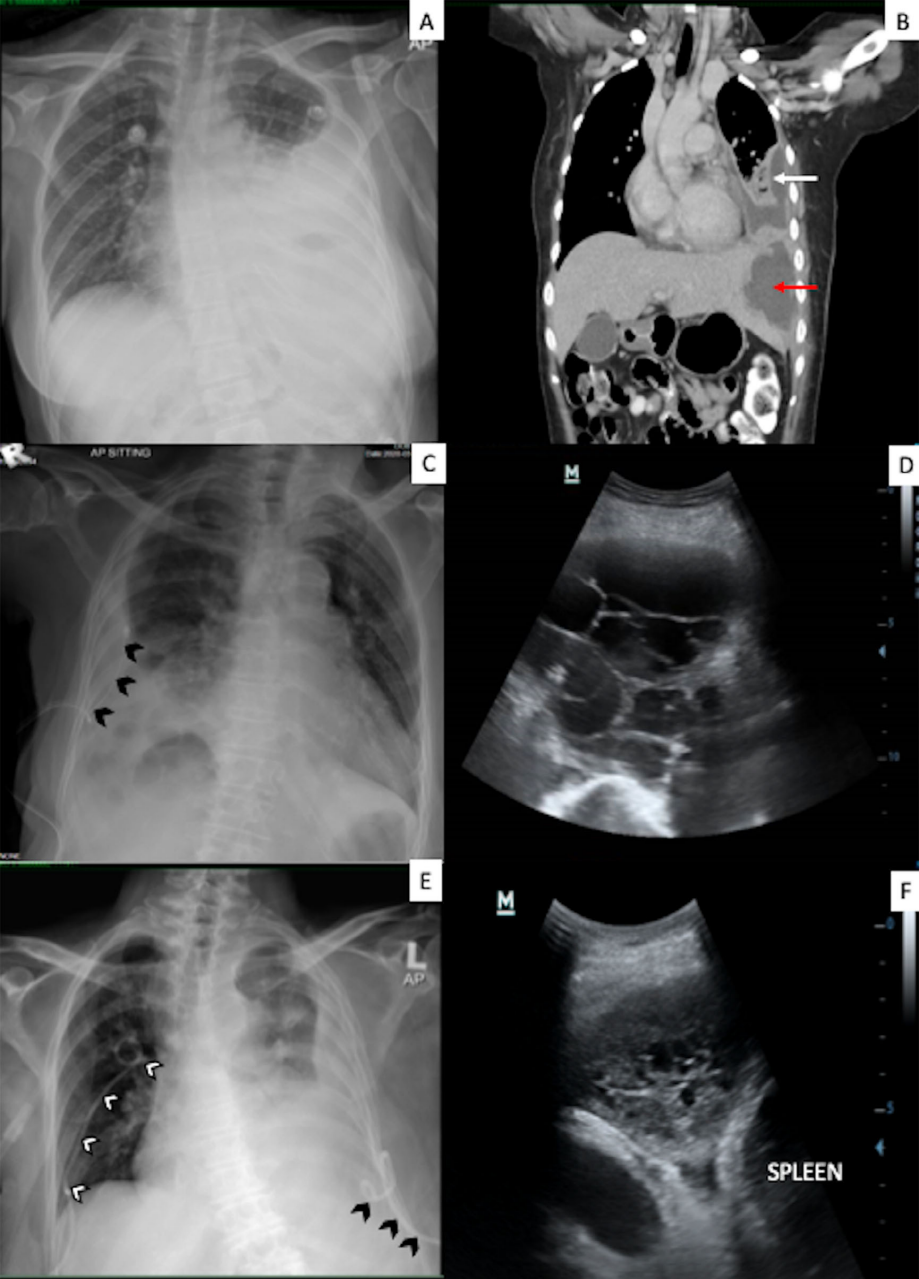
Imaging before intrapleural fibrinolysis. Patient 1: Chest radiograph showed massive left pleural effusion (A) and computed tomography (CT) (B) showed loculated left effusion (white arrow) and hepatomegaly with liver abscess (red arrow). Patient 2: Chest radiograph (C) showed moderate left effusion with chest catheter in situ (black arrows). Ultrasound of patient 2 (D) showed multiseptated effusion. Patient 3: Chest radiograph (E) showed loculated left effusion with left pigtail (black arrows) and right pigtail (white arrows) in situ. Ultrasound (F) showed complex effusion.

A 30‐year‐old woman with transfusion‐dependent β‐thalassemia and a history of splenectomy was admitted with septic shock secondary to invasive *Klebsiella pneumoniae* infection. She was started on intravenous meropenem which was de‐escalated to amoxycillin/clavulanate acid based on blood culture sensitivity. Chest radiograph revealed a moderate left pleural effusion (Fig. 1A). Computed tomography (CT) of the thorax (Fig. 1B) was suggestive of left lung empyema with liver abscess (4.9 × 6.3 cm) at segment II with subcapsular extension. Ultrasound‐guided percutaneous pigtail catheter drainage of the liver and 20 Fr chest tube inserted at left pleura cavity both drained pus which grew *K*. *pneumoniae*.

**Figure 2:**
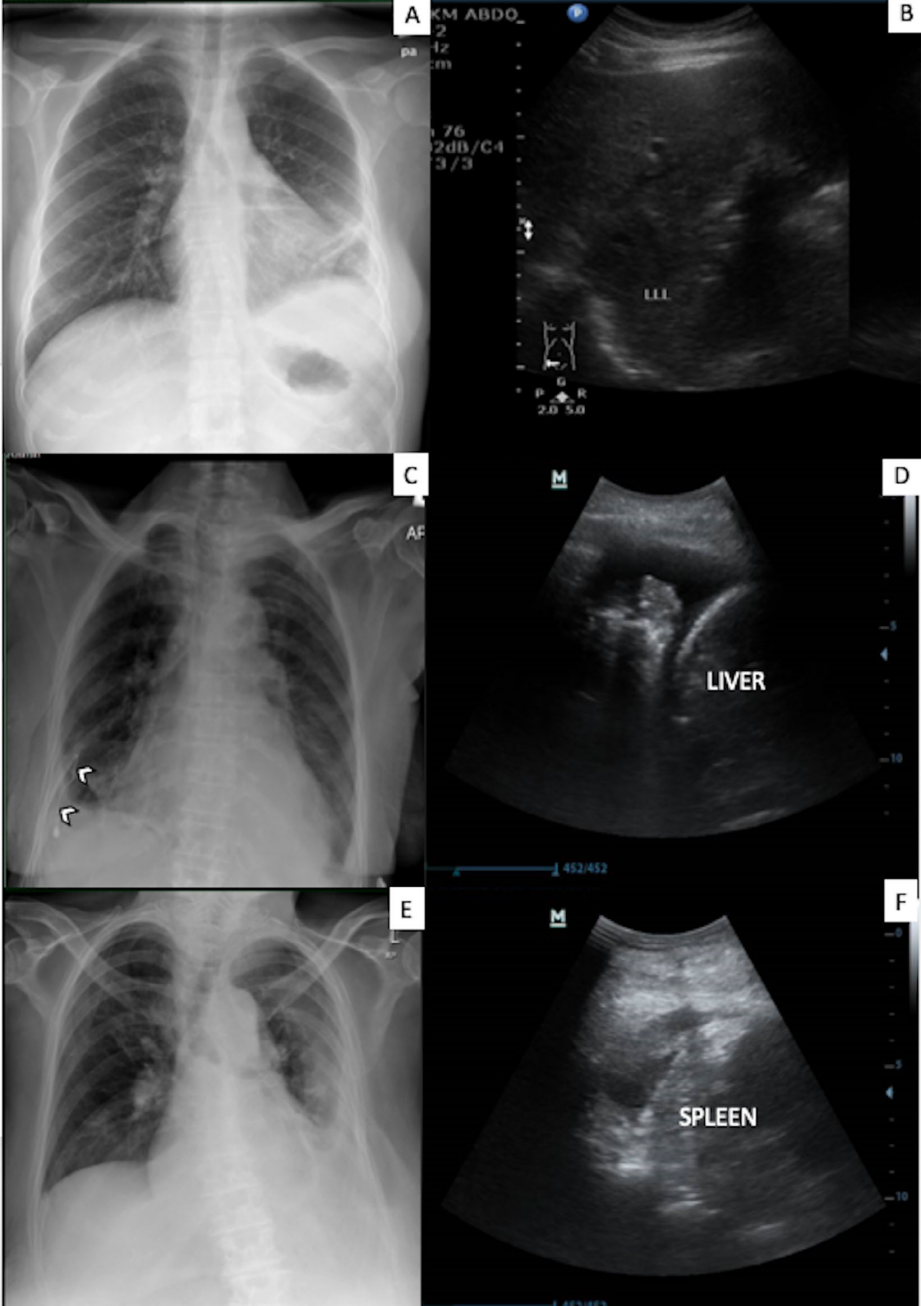
Imaging post three doses of intrapleural fibrinolysis (alteplase and deoxyribonuclease (DNase)). Patient 1: Chest radiograph (A) showed minimal left pleural effusion. Ultrasound (B) on follow‐up showed resolved liver abscess and pleural effusion. Patient 2: Chest radiograph (C) showed minimal right pleural effusion and chest catheter in situ (white arrows). Ultrasound (D) revealed unilocular effusion (compared to septated effusion before therapy). Patient 3: Chest radiograph (E) and ultrasound (F) showed minimal left effusion.

Follow‐up bedside thoracic sonography showed persistent complex pleural effusion. She received three doses of 16 mg alteplase followed by 5 mg DNase in a sequential manner 12 h apart. Her condition improved and she was successfully weaned off oxygen with improvement of pleural drainage, infective parameters (Table 1), and serial imaging (Fig. 2A, B). Haemoglobin was 7.8 g/dL prior and 8.9 g/dL after intrapleural fibrinolysis. She was discharged with oral antibiotics (tablet amoxicillin/ clavulanic acid 625 mg thrice a day). She remained well and asymptomatic with total resolution of effusion at six weeks follow‐up.

### Case 2

An 87‐year‐old woman with underlying hypertension, congestive cardiac failure, and atrial fibrillation on dabigatran presented with symptoms of dyspnoea, productive cough, and loss of appetite and weight. She received antibiotics one week prior to presentation in another centre. Chest radiograph revealed a moderate right pleural effusion (Fig. 1C) and thoracic sonography (Fig. 1D) revealed multi‐loculated effusion at the right lower hemithorax. Laboratory investigations were as shown in Table 1. An intercostal chest drain of 12 Fr was inserted targeting the biggest locule at the right posterior (R6) area, which drained 80 cc of serosanguinous fluid. Pleural fluid was exudative; however, cultures were negative. Pleural adenosine deaminase was 20 IU/L. Diagnosis of left complex parapneumonic effusion was made. Intravenous amoxicillin/clavulanic acid was commenced during hospitalization.

We then decided for IPFT, dabigatran was withheld one day earlier, and three doses of sequential 16 mg alteplase and 5 mg DNase were given as per our protocol. Following that, there was remarkable improvement of pleural drainage (Table 1).

The patient's general condition and cough improved and she remained afebrile. Serial imaging showed improvement (Fig. 2C, D). Dabigatran was resumed 24 h after the completion of intrapleural fibrinolysis without immediate complications. She was discharged with oral antibiotics (tablet amoxicillin/clavulanic acid 625 mg twice a day) for another four weeks.

### Case 3

An 80‐year‐old lady with underlying type 2 diabetes mellitus and hypertension presented with a left neck swelling and bilateral effusion. She had undergone left thoracentesis and antibiotic treatment three weeks earlier at a different centre. In our centre, bilateral pigtail catheters were inserted.

Laboratory findings were as per Table 1. Pleural fluid cytology and excision biopsy of cervical nodes revealed diffuse B‐cell lymphoma. She was given the first cycle of chemotherapy R‐mini CHOP regimen (rituximab, cyclophosphamide, vincristine, prednisolone).

The right lung was fully expanded. However, the patient developed fever in the ward with raised C‐reactive protein and chest radiograph (Fig. 1E) showed a loculated left pleural effusion. Thoracic sonography (Fig. 1F) confirmed the presence of septated effusion. Pleural drainage was minimal. Diagnosis of left parapneumonic effusion was made (in the background of lymphomatous effusion). Intravenous cefepime 2 g thrice daily was commenced and decision for IPFT was made in view of poor chest drainage.

She received three doses of 16 mg intrapleural alteplase with 5 mg intrapleural DNase as per protocol. There was subsequent improvement of pleural drainage and her fever subsided. There was minimal drop of haemoglobin from 8.6 to 7.8 g/dL with no immediate bleeding complications throughout hospitalization.

She continued to improve clinically and serial imaging also showed improvement (Fig. 2E, F). She was discharged well.

## Discussion

The treatment of pleural infection involves simultaneous empirical broad‐spectrum antibiotic therapy and drainage of infected pleural fluid. However, unsatisfactory drainage can occur due to septations or loculated effusion. The use of intrapleural fibrinolytic therapy has been recognized as an alternative treatment to surgical intervention [4]. This paradigm shift of a less invasive IPFT to facilitate pleural fluid drainage in patients high risk for surgery avoids the need for surgical intervention. MIST‐2 and a ‘real‐life’ study by Piccolo et al. have shown significant improvement in pleural drainage with the combined use of intrapleural recombinant human tissue plasminogen activator and recombinant human DNase, with more than 90% patients avoiding surgery intervention [3, 5].

Our patients presented with complex pleural effusion with poor drainage from intercostal chest catheter. Two of the patients were in the advanced age group (more than 80 years old) who were at high risk for surgical intervention. All patients were successfully treated with less invasive intervention by IPFT. We still do not know the optimal dosing regimen and protocol for IPFT [6]. The regimen of 10 mg alteplase and 5 mg DNase twice per day that was used in MIST‐2 is notably still an empiric choice [3]. The study by Abu‐Daff et al. has shown the effectiveness of intrapleural 16 mg alteplase alone in parapneumonic effusion or empyema [7]. We chose 16 mg alteplase dose (with supplementary 5 mg DNase) to simplify its dispensing from the pharmacy in the frozen form as the ampoule contains 50 mg and should be utilized within 24 h. Our patients were given a total of three doses of combined intrapleural alteplase and DNase in a short duration of 24 h. Pleural fluid drainage was as effective as the conventional three‐day regimen in MIST‐2. This method reduces the duration of chest drain in pleural space and hospital stay. The modified regimen in using 16 mg alteplase is by case to case basis and dependent on the alteplase drug formulation.

Bleeding risk following IPFT instillation is a concern for many clinicians. Our three patients had mild anaemia and bleeding risk at baseline. One patient was on oral anticoagulant for atrial fibrillation which was withheld prior to IPFT. We suggest anticoagulation to be withheld based on its half‐life. In all our patients, there were no immediate bleeding events and haemoglobin remained static after IPFT in all cases. This demonstrates the safety of intrapleural fibrinolysis in patients with mild anaemia and bleeding risk. Intravenous alteplase has low rates of severe bleeding of 0.5–8.5% [8]. Although the mechanism of alteplase in pleural space remains unclear, it is safe to be used intrapleurally and rarely causes systemic haemorrhage. Pleural bleeding and bleeding from chest tube sites have been reported at 1.8–6% [3, 5, 9].

A treatment shift to a less invasive approach with IPFT appears promising with good safety profile. Our case series of complex pleural effusion successfully treated with short‐duration intrapleural alteplase and DNase demonstrates the safety of intrapleural fibrinolysis in patients with bleeding risk and anaemia using our modified regimen of 16 mg alteplase and 5 mg DNase. We believe this can be an alternative regimen in centres with different alteplase formulation.

### Disclosure Statement

Appropriate written informed consent was obtained for publication of this case series and accompanying images. We received fundamental grant for a study entitled “The Effectiveness of Short Duration High Intensity Intrapleural Alteplase with DNase (Pulmozyme) in the Management of Pleural Infection” which was approved by UKM Ethics Committee (FF‐2020‐008).
